# Impact of extracorporeal membrane oxygenation on voriconazole plasma concentrations: A retrospective study

**DOI:** 10.3389/fphar.2022.972585

**Published:** 2022-08-17

**Authors:** Qinghua Ye, Xin Yu, Wenqian Chen, Min Li, Sichao Gu, Linna Huang, Qingyuan Zhan, Chen Wang

**Affiliations:** ^1^ Peking University China-Japan Friendship School of Clinical Medicine, Beijing, China; ^2^ Department of Pulmonary and Critical Care Medicine, Center of Respiratory Medicine, China-Japan Friendship Hospital, National Clinical Research Center for Respiratory Diseases, Beijing, China; ^3^ Department of Pharmacy, China-Japan Friendship Hospital, Beijing, China; ^4^ Chinese Academy of Medical Sciences and Peking Union Medical College, Beijing, China

**Keywords:** voriconazole concentrations, extracorporeal membrane oxygenation, therapeutic drug monitoring, pharmacokinetics, critically ill patients

## Abstract

**Aims:** We aimed to assess the impact of extracorporeal membrane oxygenation (ECMO) on voriconazole exposure.

**Methods:** Adult critically ill patients with or without ECMO support receiving intravenous voriconazole therapy were included in this retrospective study conducted in a tertiary referral intensive care unit. The first therapeutic drug monitoring (TDM) results of voriconazole in ECMO patients and non-ECMO patients were collected, and the prevalence of subtherapeutic concentrations was analyzed. Multivariate analyses were performed to evaluate the effect of ECMO on voriconazole exposure.

**Results:** A total of 132 patients (including 66 patients with ECMO support) were enrolled and their respective first voriconazole trough concentrations (C_min_) were recorded. The median C_min_ of the ECMO group and the non-ECMO group was 1.9 (1.4–4.4) and 4.4 (3.2–6.9) mg/L, respectively (*p* = 0.000), and the proportion of the two groups in subtherapeutic concentrations range (<2 mg/L) was 51.5% and 7.6%, respectively (*p* = 0.000). Multiple linear regression analysis of voriconazole C_min_ identified that the use of ECMO and coadministration of glucocorticoids were associated with significantly reduced concentrations, while increasing SOFA score and increasing daily dose were associated with significantly increased concentrations. The model accounted for 32.2% of the variability of voriconazole C_min_. Furthermore, binary logistic regression demonstrated that the use of ECMO was an independent risk factor (OR = 7.78, *p* = 0.012) for insufficient voriconazole exposure.

**Conclusion:** Our findings showed that, in addition to the known drug interactions, ECMO is a significant covariable affecting voriconazole exposure. In addition, SOFA score was identified as a factor associated with increased voriconazole concentration.

## Introduction

Invasive fungal infections are life-threatening diseases associated with considerable morbidity and mortality (Chamilos et al., 2006; Bassetti and Bouza, 2017; Sanguinetti et al., 2019). Although these infections typically occur in the immunocompromised host, invasive pulmonary aspergillosis has been increasingly reported in critically ill patients even among immunocompetent patients (Tudesq et al., 2019). More recently, severe viral infection, including influenza and COVID-19, have also been identified as risk factors for IPA in critically ill patients, and is associated with increased mortality (Schauwvlieghe et al., 2018; Dellière et al., 2020; Verweij et al., 2020; Bartoletti et al., 2021; Mitaka et al., 2021).

Voriconazole, a second-generation triazole antifungal agent with a broad-spectrum of activity, is considered a first-line drug for the treatment of invasive aspergillosis (Patterson et al., 2016). In recent years, numerous studies have investigated the exposure–response relationship of voriconazole. The findings from these studies suggested that high concentrations might be associated with clinical adverse events, whilst inadequate concentrations were more likely to lead to treatment failure (Dolton and McLachlan, 2014; Jin et al., 2016). At present, the target trough concentration (C_min_) recommended by the guideline is higher than 1–2 mg/L and lower than 5–6 mg/L (Ashbee et al., 2014; Patterson et al., 2016). However, in daily practice, the concentration of voriconazole varies greatly not only between patients, but also within individual patients over time (Mangal et al., 2018). Voriconazole is metabolized by enzymes that predominantly include CYP2C19, CYP3A4, and CYP2C9, and the concomitant use of inducers or inhibitors of these drug-metabolizing enzymes can affect voriconazole exposure (Blanco-Dorado et al., 2020; Jia et al., 2021; Tian et al., 2021). In addition, other factors, including body weight, the nonlinear pharmacokinetic properties, the cytochrome P450 2C19 genotype, liver function and inflammation, were also reported to be responsible for the large differences in voriconazole exposure (Shi et al., 2019). To ensure the therapeutic concentrations of voriconazole are reached clinically, therapeutic drug monitoring (TDM), which relies on the attainment of steady state pharmacokinetics, is widely used. However, the waiting period to reach steady-state (usually 5–7 days) is particularly problematic in critically ill patients and may contribute to a worse prognosis (Mangal et al., 2018). Therefore, the identification of factors affecting voriconazole exposure is important to optimize its clinical application.

In the last decade, the use of extracorporeal membrane oxygenation (ECMO) as a life-support modality used in patients with refractory cardiac and/or respiratory failure, has significantly increased in the adult population (Brodie et al., 2019). Patients with ECMO support are usually the most seriously ill in intensive care units, who are often receiving broad-spectrum antibiotics and have multiple pathogens entry points. These factors are thought to predispose them to fungal infection, which is associated with poor prognosis (Cavayas et al., 2018; Poth et al., 2022). Since ECMO itself is not a disease-modifying intervention, treatment outcomes of patients with fungal infection on ECMO will largely rely on whether adequate antifungal therapy is provided. Unfortunately, multiple studies have suggested that the presence of ECMO will significantly change the pharmacokinetics (PK) of antimicrobial agents due to a larger volume of distribution, caused by circuit sequestration and hemodilution from priming solutions (Duceppe et al., 2021). Voriconazole is a lipophilic and highly protein-bound drug for which its significant sequestration in ECMO circuits seems to be expected. Data from case-reports and *ex vivo* studies corroborate this insight. The *ex vivo* studies demonstrated significant voriconazole losses in ECMO circuits, even up to 80% in some reports (Mehta et al., 2007; Cies et al., 2020; Raffaeli et al., 2020; Zhang et al., 2021b). Consistent with these findings, case reports reported empirical use of higher doses, subtherapeutic or undetectable trough concentrations and/or the need for frequent dose increases of voriconazole in patients during ECMO support (Brüggemann et al., 2008; Ruiz et al., 2009; Spriet et al., 2009; Winiszewski et al., 2018; Mathieu et al., 2021; Peterson et al., 2021). Moreover, Spriet et al (2009) proposed the saturation theory of circuit’s binding sites. They increased the dose of voriconazole at the beginning of ECMO therapy to avoid subtherapeutic concentration, but a few days later, they observed drug accumulation. In summary, in order to compensate for the expected loss in the ECMO circuit, it appears necessary to increase the voriconazole dose in advance before ECMO initiation or during ECMO support. However, a recently published study by Daele et al. refutes this idea (Van Daele et al., 2021). In this study, the dose and concentration of voriconazole were similar on ECMO and non-ECMO days; neither the presence of ECMO nor the duration of ECMO on sampling day were significant covariates affecting voriconazole exposure. Therefore, more studies are needed to demonstrate the real impact of ECMO on voriconazole exposure.

The objective of this study was to evaluate the effect of ECMO on voriconazole plasma exposure by comparing the differences of voriconazole C_min_ and relevant clinical variables between patients with and without ECMO support, which contributed to optimizing the clinical application of voriconazole in patients with invasive aspergillosis and other fungal infections requiring ECMO support.

## Methods

### Study design and patients

This retrospective, observational cohort study was conducted in the respiratory intensive care unit of the China-Japan Friendship Hospital, National Clinical Research Center for Respiratory Diseases, a 1600-bed teaching hospital in Beijing. All adult patients (≥ 18 years old) hospitalized in the ICU from August 2017 to December 2021, who were treated with intravenous voriconazole for possible, probable or proven *Aspergillus* infection (De Pauw et al., 2008) and had at least one voriconazole C_min_ available during treatment were eligible for the inclusion criteria. Patients who received ECMO support during at least part of voriconazole treatment and had available C_min_ during ECMO support were assigned to the ECMO group. As a control, patients who had never received ECMO support during ICU hospitalization, or voriconazole therapy and its TDM were conducted during non-ECMO periods (including before ECMO initiation and 5 days after weaning from ECMO) were enrolled in the non-ECMO group. We only collected the patient’s first voriconazole C_min_ for analysis, and for patients in the ECMO group, this concentration refers to the first TDM results during ECMO support. This study was approved and supervised by the Ethics Committee of China-Japan Friendship Hospital (2021-134-K92), and written informed consent was waived.

From the electronic medical records, the patient’s demographic characteristics and clinical data on the sampling day were collected, including gender, age, body weight, the severity of illness (APACHE II and SOFA scores), voriconazole daily dose previous 24 h on sampling day, voriconazole C_min_, relevant laboratory test results (liver function, renal function and procalcitonin), the use of continuous renal replacement therapy (CRRT) and ECMO. Interacting drugs commonly used in our center that may affect voriconazole plasma exposure were also recorded, including proton pump inhibitors (PPIs), calcineurin inhibitors (CNIs), glucocorticoids, analgesic and sedative drugs (including fentanyl and midazolam) were also recorded. A standard case report form was used during the study.

### Voriconazole administration and trough concentration measurement

Among critically ill patients in our hospital, the TDM of voriconazole is routinely performed, usually 5–7 days after administration. Nurses would collect the blood sample within half an hour before the subsequent administration under steady-state conditions. Voriconazole C_min_ was measured by an ultra-high-performance liquid chromatograph-tandem mass spectrometry method (UPLC-MS/MS; Waters, United States). According to the guideline recommendations (Patterson et al., 2016), voriconazole was administered as an initial loading dose of 6 mg/kg i.v., every 12 h on day 1, followed by 4 mg/kg i.v., every 12 h for maintenance. Thereafter, the subsequent dose was adjusted by the attending physician based on clinical reactions and TDM results. Most guidelines recommend a lower target value of 1–2 mg/L, with higher targets (> 2 mg/L) for severe infections and diseases with poor prognosis, which may be appropriate for critically ill patients (Miyakis et al., 2010; Ashbee et al., 2014), so we define a lower limit of C_min_ as > 2 mg/L. There are currently no voriconazole dosing recommendations for the ECMO patient population, and we did not empirically use higher doses in this patient population.

### Extracorporeal circuits

The mode and settings of ECMO were determined based on the clinical context. ECMO circuit consisted of polyvinyl chloride tubing, a polymethyl-pentene membrane oxygenator, a blood pump, and a heat exchanger. The ECMO circuit was primed with 600 ml of normal saline. The type of ECMO equipment, the mode of ECMO and the running days of ECMO when collecting voriconazole blood samples were recorded. Considering that new binding sites were presumed to be available each time the ECMO circuit was changed, the running time was reset to day 1 (Van Daele et al., 2021).

### Statistical analysis

Statistical analysis was conducted using SPSS version 25.0 (IBM Corp., Armonk, NY). Categorical data are presented as frequencies (%), and continuous data are presented as the means ± standard deviations (SD) or medians (interquartile range, IQR). Student’s t-tests or Mann-Whitney U tests were used to analyze continuous variables between groups, and categorical variables were analyzed by the Chi-square test or Fisher’s exact test. A two-tailed *p* value < 0.05 was considered statistically significant. The relationship between initial voriconazole levels and relevant continuous variables was examined using the Spearman correlation coefficient. Variables that were considered clinically relevant and statistically significant in univariate analysis were entered into the multivariate model. The determinations of voriconazole trough concentration were analyzed using multiple linear regression. The predictors of voriconazole subtherapeutic concentration were analyzed by logistic regression.

## Results

### Patient characteristics

A total of 132 critically ill adult patients were included in this study, including 66 patients who received ECMO support during at least part of voriconazole treatment. In the non-ECMO group, 10 patients received ECMO support before or after voriconazole therapy, of which 7 patients’ TDM results were obtained before ECMO initiation and the remaining three patients were obtained after weaning from ECMO (post-ECMO 5–7 days). Daily maintenance doses of voriconazole in all patients before sampling ranged from 200 to 800 mg, mostly 400 mg (93.9%). The mean daily maintenance dose per kg of body weight for all patients was 6.3 ± 1.4 mg/kg. Key demographics, clinical characteristics, and drug combinations are presented in Table 1. In this study, 78.8% of patients (*n* = 104) had concomitant medications with PPIs, most of them omeprazole and pantoprazole. With respect to glucocorticoids, its use was recorded in 64 of 132 patients (48.5%), most of them methylprednisolone (both intravenous and oral routes). We compared the distribution of various characteristics between the ECMO group and the non-ECMO group in Table 1. Compared with the non-ECMO group, the patients in the ECMO group were younger (52.0 ± 17.2 vs. 64.1 ± 14.5; *p* = 0.000), with higher SOFA scores (10.3 ± 3.4 vs. 8.4 ± 3.8; *p* = 0.003) and higher albumin levels (38.4 ± 6.5 vs. 32.9 ± 4.2; *p* = 0.000). Furthermore, a higher proportion of patients in the ECMO group received concomitant administration with PPIs (97.0% vs. 60.6; *p* = 0.000) and glucocorticoids (65.2% vs. 31.8%; *p* = 0.000).

**TABLE 1:** Comparison of clinical characteristics between ECMO group and non-ECMO group.

Parameter	Total	ECMO	Non-ECMO	*p*-value
	*n* = 132	*n* = 66	*n* = 66	
Age (years)	58.1 ± 17.0	52.0 ± 17.2	64.1 ± 14.5	0.000
Female	39 (29.5)	18 (27.3)	21 (31.8)	0.567
Total body weight (kg)	66.3 ± 15.6	68.9 ± 16.9	63.8 ± 13.9	0.060
APACHE II	22.9 ± 5.3	22.2 ± 4.6	23.7 ± 5.9	0.111
SOFA	9.4 ± 3.7	10.3 ± 3.4	8.4 ± 3.8	0.003
ALT (U/L)	29 (16–62)	26 (17–58.5)	30 (15.8–64.3)	0.804
AST (U/L)	37 (26–68)	37 (26.5–71)	41 (24.8–60.3)	0.936
TBIL (umol/L)	13.7 (8.3–26.3)	19.3 (10.7–31.8)	10.4 (7.1–17.0)	0.057
DBIL (umol/L)	6.2 (3.1–14.0)	7.2 (4.6–16.8)	5.6 (2.6–9.3)	0.091
ALB (g/L)	35.5 ± 6.0	38.4 ± 6.5	32.9 ± 4.2	0.000
Urea (mmol/L)	10.4 (6.7–16.6)	11.1 (6.8–17.1)	10.1 (5.8–15.2)	0.428
SCR (umol/L)	75.7 (50–142.0)	63.3 (43.9–134.9)	85.1 (56.8–144.3)	0.163
PCT (ng/ml)	0.8 (0.2–3.2)	1.1 (0.3–4.5)	0.6 (0.2–2.8)	0.297
CRRT	43 (32.6)	21 (31.8)	22 (33.3)	0.853
Previous daily dose (mg/kg)	6.3 ± 1.4	6.0 ± 1.4	6.7 ± 1.4	0.004
C_min_ (mg/L)	3.6 (1.8–5.3)	1.9 (1.4–4.4)	4.4 (3.2–6.9)	0.000
C_min_/dose	0.57 (0.33–0.89)	0.40 (0.22–0.69)	0.69 (0.47–0.97)	0.001
Subtherapeutic C_min_ (<2 mg/L)	39 (29.5)	34 (51.5)	5 (7.6)	0.000
Supratherapeutic C_min_ (>5.5 mg/L)	30 (22.7)	8 (12.1)	22 (33.3)	0.004
Concomitant medication				
Calcineurin inhibitors	18 (13.6)	11 (16.7)	7 (10.6)	0.310
Proton pump inhibitors	104 (78.8)	64 (97.0)	40 (60.6)	0.000
Glucocorticoids	64 (48.5)	43 (65.2)	21 (31.8)	0.000
Others^a^	50 (37.9)	23 (34.8)	27 (40.9)	0.473
Mortality	64 (48.5)	31 (47.0)	33 (50.0)	0.728

a Including fentanyl and midazolam.

Note: Data are presented as n (%), mean ± SD, or median (IQR). Abbreviations: ECMO, extracorporeal membrane oxygenation; APACHE II, acute physiology and chronic health evaluation II; SOFA, sequential organ failure assessment; ALT, alanine aminotransferase; AST, aspartate aminotransferase; TBIL, total bilirubin; DBIL, direct bilirubin; ALB, albumin; SCR, serum creatinine; PCT, procalcitonin; CRRT, continuous renal replacement therapy; C_min_, voriconazole trough concentration.

### Extracorporeal circuits

Data concerning ECMO was summarized in Table 2. Most patients (93.9%) used veno-venous (VV)-ECMO on the sampling day, and the remaining patients (6.1%) used veno-arterial (VA)-ECMO. The mean ECMO blood flow rate on the sampling day was 3.77 ± 0.67 L/min, and the median duration of ECMO on the sampling day was 5 (IQR, 4–11) days. 31.8% of patients (*n* = 21) in the ECMO group concurrently received renal replacement therapy. Eight patients changed the membrane oxygenators during ECMO support.

**TABLE 2:** ECMO circuits.

Parameters	Value
ECMO mode	
VV-ECMO	62 (93.9)
VA-ECMO	4 (6.1)
ECMO apparatus	
Maquet (BE-PLS 2050; Germany)	61 (92.4)
Sorin (D905, Italy)	5 (7.6)
Blood flow rate (L/min)	3.77 ± 0.67
ECMO duration on sampling day (days)	5 (4–11)
ECMO duration (days)	13 (8–22)
At least one of ECMO components change	8 (12.1)
CRRT	21 (31.8)

Note: Data are presented as n (%), mean ± SD, or median (IQR). Abbreviations: ECMO, extracorporeal membrane oxygenation; VV-ECMO, veno-venous ECMO; VA-ECMO, veno-arterial ECMO; CRRT, continuous renal replacement therapy.

### Voriconazole trough concentrations

**FIGURE 1:**
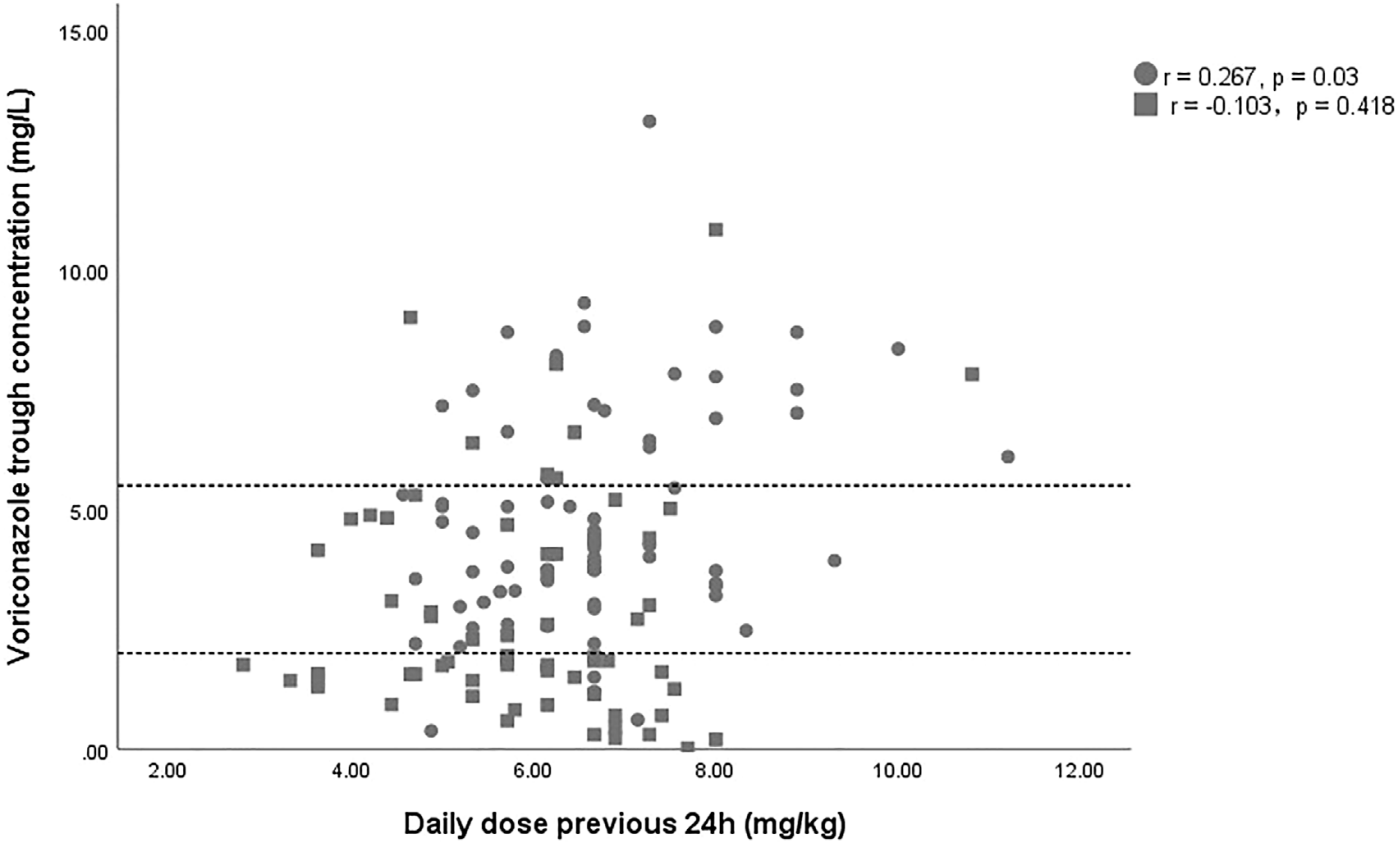
Scatterplot of the voriconazole trough concentration versus the voriconazole dose. The square and circles represent data of patient with and without ECMO support. The area between the dashed lines indicates the therapeutic range (2–5.5 mg/L).

**FIGURE 2:**
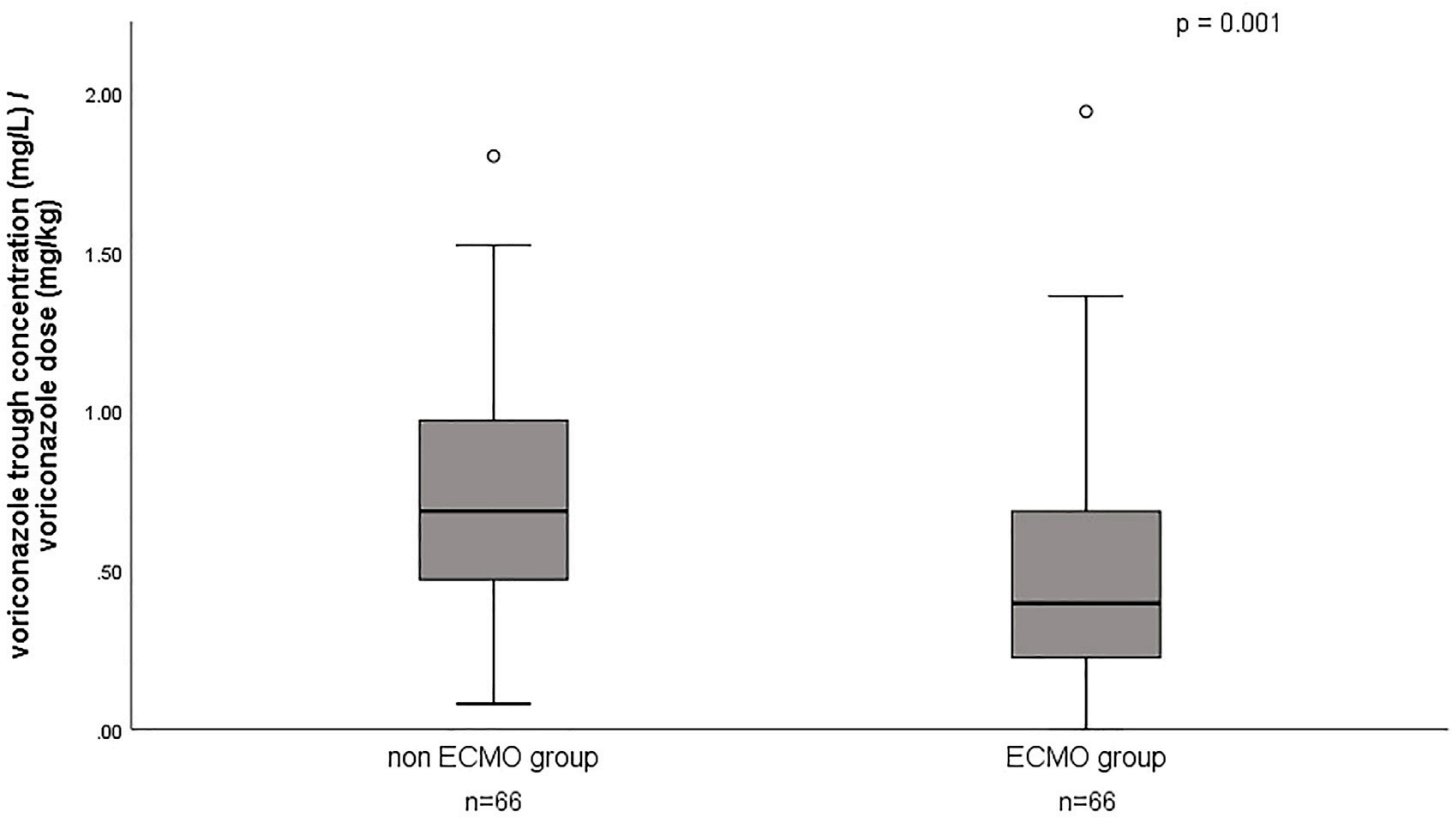
Effect of ECMO on dose-normalized voriconazole trough concentrations.

A total of 132 voriconazole C_min_ were included in this study, with a median C_min_ of 3.6 (IQR 1.8–5.3) mg/L, and 29.5% of the patients were in subtherapeutic concentration (<2 mg/L) (Table 1). Figure 1 shows the distribution of measured voriconazole C_min_ and their respective doses. Compared with the non-ECMO group, both the C_min_ and the dose-normalized C_min_ were significantly lower [C_min_, 1.9 (1.4–4.4) vs. 4.4 (3.2–6.9), *p* = 0.000; C_min_/dose, 0.40 (0.22–0.69) vs. 0.69 (0.47–0.97), *p* = 0.001] in the ECMO group, while the proportion in subtherapeutic concentration range was significantly higher (51.5% vs. 7.6%; *p* = 0.000). The effect of ECMO on dose-normalized C_min_ was shown in Figure 2. Furthermore, extensive inter-individual variability was observed in voriconazole C_min_ in the ECMO group, ranging from 0.01 to 10.84 mg/L, with a coefficient of variation (CV) of 79.3%.

### Factors affecting voriconazole trough concentrations

Differences in clinical variables between patients with subtherapeutic concentrations (< 2 mg/L) and those with therapeutic or supratherapeutic trough concentrations (> 2 mg/L) were shown in Table 3. Multivariate analysis by linear regression was performed to identify the determinants of voriconazole C_min_ (Table 4). Increasing SOFA scores and increasing daily dose were associated with significantly increased voriconazole concentrations; factors associated with reduced voriconazole concentrations included the use of ECMO and coadministration with glucocorticoids. We also conducted a binary logistic regression analysis to identify the predictors of voriconazole insufficient exposure (Table 5). The results showed that only the use of ECMO was an independent risk factor for voriconazole subtherapeutic concentration (OR 7.783, *p* = 0.012).

**TABLE 3:** Univariate analysis for different voriconazole concentration ranges.

Parameter	By voriconazole trough concentrations group		*p*-value
	<2 mg/L, *n* = 39	>2 mg/L, *n* = 93	
Female	11 (28.2)	28 (30.1)	0.827
Age (years)	53.2 ± 17.0	60.1 ± 16.6	0.031
Total body weight (kg)	67.3 ± 14.6	65.9 ± 16.1	0.627
APACHE II	21.6 ± 4.2	23.5 ± 5.7	0.039
SOFA	9.2 ± 3.4	9.4 ± 3.9	0.805
Previous daily dose (mg/kg)	6.0 ± 1.3	6.5 ± 1.4	0.066
ALT (U/L)	39 (18–61)	27 (14.5–63)	0.645
AST (U/L)	35.5 (24.3–60)	39 (26.5–75)	0.551
TBIL (umol/L)	12.8 (8.2–27.9)	13.8 (8.3–26.9)	0.668
DBIL (umol/L)	5.2 (2.8–15.4)	6.9 (3.7–13.8)	0.543
ALB (g/L)	38.1 ± 6.8	34.6 ± 5.5	0.023
SCR (umol/L)	48.7 (41.3–94.1)	86.1 (58.8–146.7)	0.027
PCT (ng/ml)	0.6 (0.2–3.1)	1.1 (0.3–3.2)	0.561
Concomitant medication			
Calcineurin inhibitors	8 (20.5)	10 (10.8)	0.136
Proton pump inhibitors	35 (89.7)	69 (74.2)	0.046
Glucocorticoid	29 (74.4)	35 (37.6)	0.000
Others^a^	16 (41.0)	34 (36.6)	0.629
CRRT	7 (17.9)	36 (38.7)	0.02
ECMO	34 (87.2)	32 (34.4)	0.000

a Including fentanyl and midazolam.

Note: Data are presented as n (%), mean ± SD, or median (IQR). Abbreviations: APACHE II, acute physiology and chronic health evaluation II; SOFA, sequential organ failure assessment; ALT, alanine aminotransferase; AST, aspartate aminotransferase; TBIL, total bilirubin; DBIL, direct bilirubin; ALB, albumin; SCR, serum creatinine; PCT, procalcitonin; CRRT, continuous renal replacement therapy; ECMO, extracorporeal membrane oxygenation.

**TABLE 4:** Multiple linear regression analysis of voriconazole trough concentration determinants.

	Coefficient	*t*	*p*-value	VIF
Age	0.018	1.334	0.185	1.391
APACHE II	0.002	0.038	0.970	2.585
SOFA	0.201	2.237	0.027	2.808
SCR	0.002	0.829	0.409	1.520
Dose before sampling	0.403	2.498	0.014	1.355
Proton pump inhibitors	0.662	1.060	0.291	1.755
Glucocorticoids	−1.608	−3.164	0.002	1.707
CRRT	−0.553	−1.048	0.297	1.615
ECMO	−1.463	−2.752	0.007	1.871
Constant value	−0.650	−0.414	0.680	
F		6.287		
*p*		0.000		
*R* ^2^		0.322		

Dependent variable: voriconazole trough concentration. VIF, variance inflation factor. Abbreviations: APACHE II, acute physiology and chronic health evaluation II; SOFA, sequential organ failure assessment; SCR, serum creatinine; CRRT, continuous renal replacement therapy; ECMO, extracorporeal membrane oxygenation.

**TABLE 5:** Binary logistic regression analysis for subtherapeutic trough concentration (< 2 mg/L).

Variables	Univariable	*p*-value	Multivariable	*p*-value
	OR (95% CI)		OR (95% CI)	
Age	1.024 (1.002–1.047)	0.035	1.022 (0.983–1.063)	0.269
APACHE II	1.072 (0.995–1.156)	0.068	1.037 (0.916–1.174)	0.566
SOFA	1.013 (0.916–1.120)	0.803		
ALB	0.909 (0.842–0.981)	0.014	0.972 (0.880–1.074)	0.577
SCR	1.007 (1.001–1.013)	0.034	1.010 (0.998–1.023)	0.097
Dose before sampling	1.310 (0.979–1.753)	0.069	1.407 (0.852–2.323)	0.182
Proton pump inhibitors	3.043 (0.979–9.459)	0.054	0.573 (0.071–4.639)	0.602
Glucocorticoids	4.806 (2.091–11.046)	0.000	2.201 (0.465–10.421)	0.320
CRRT	0.346 (0.138–0.867)	0.024	0.523 (0.116–2.354)	0.399
ECMO	12.962 (4.620–36.369)	0.000	7.783 (1.557–35.008)	0.012

Abbreviations: APACHE II, acute physiology and chronic health evaluation II; SOFA, sequential organ failure assessment; ALB, albumin; SCR, serum creatinine; CRRT, continuous renal replacement therapy; ECMO, extracorporeal membrane oxygenation.

**FIGURE 3:**
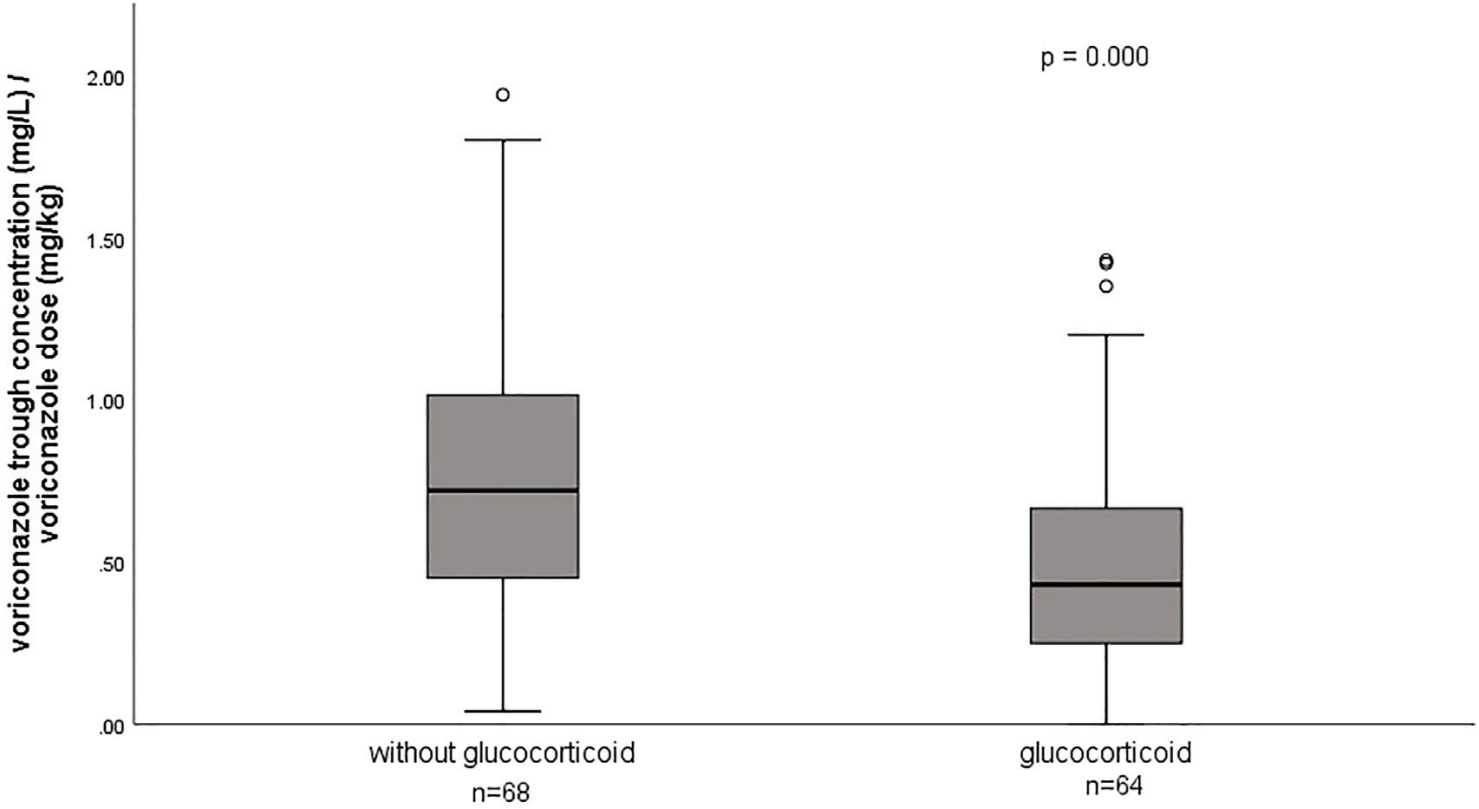
Effect of glucocorticoid on dose-normalized voriconazole trough concentrations.

**FIGURE 4:**
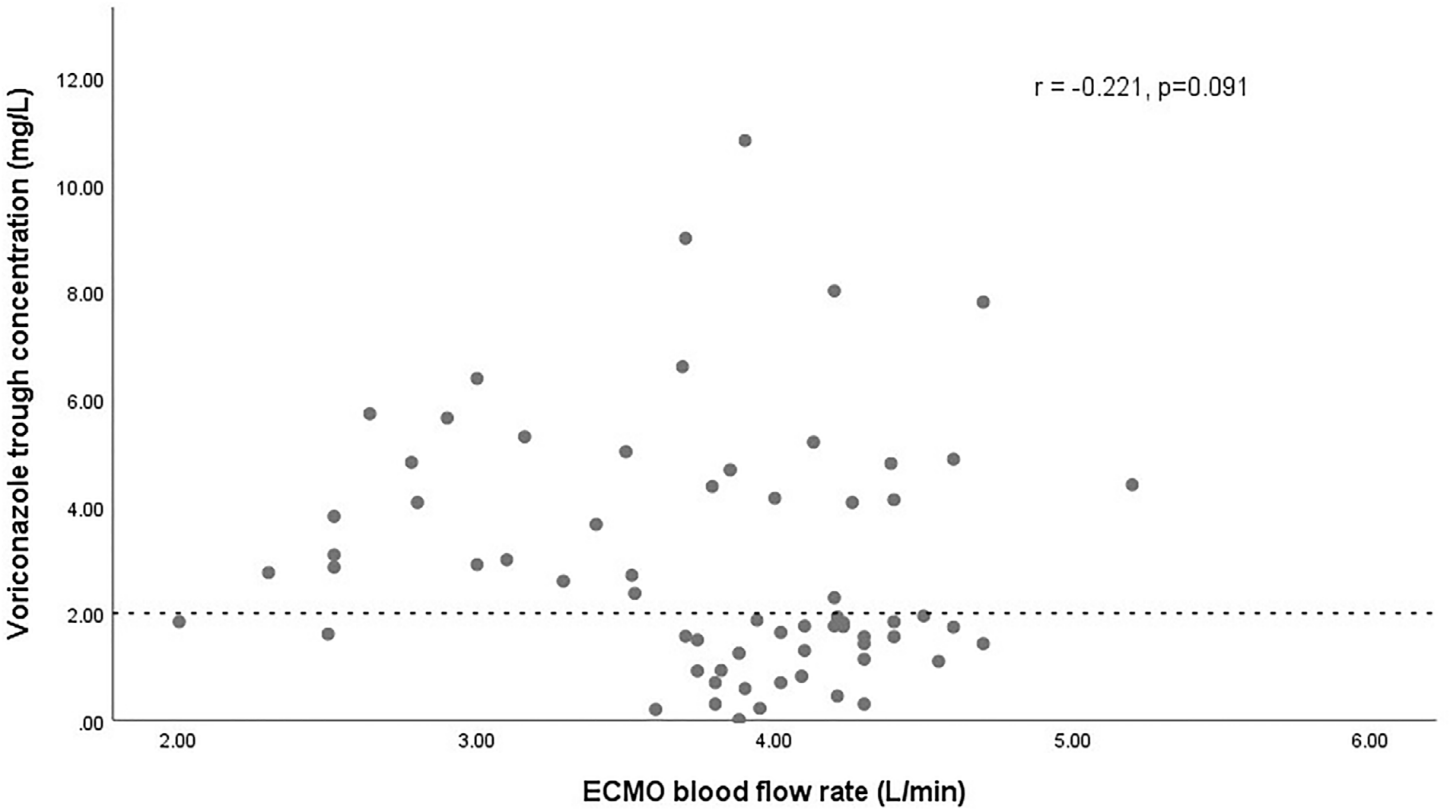
Scatterplot of the voriconazole trough concentrations versus the ECMO blood flow rate with spearman-coefficient and *p*-value (*n* = 66). The dashed line represents the lower limit concentration of 2 mg/L.

**FIGURE 5:**
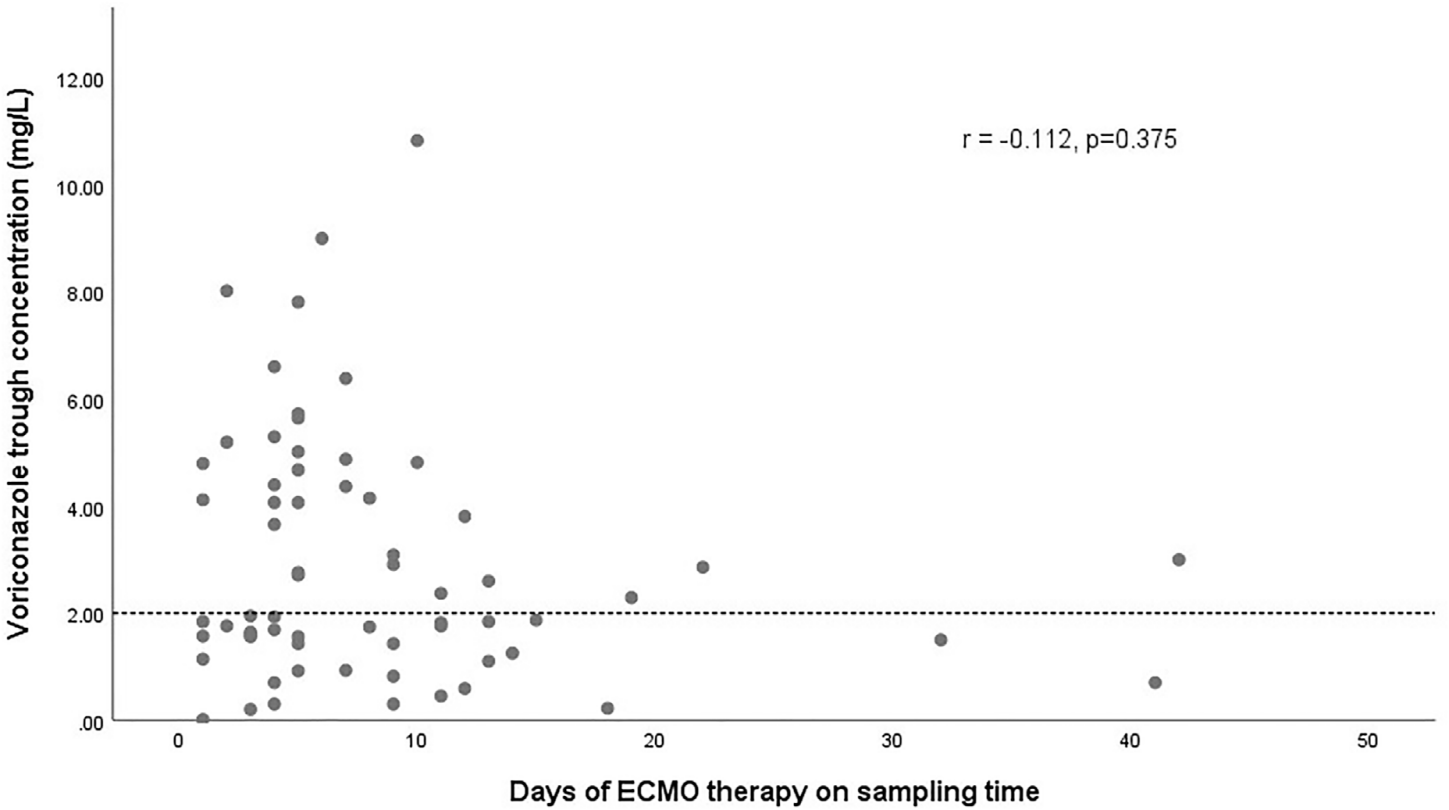
Scatterplot of voriconazole trough concentrations versus the duration of ECMO on sampling day with spearman-coefficient and *p*-value (*n* = 66). The dashed line represents the lower limit concentration of 2 mg/L.

The effect of glucocorticoids on dose-normalized C_min_ was shown in Figure 3. Figure 4 shows that there seemed to be a negative correlation between voriconazole C_min_ and the ECMO blood flow rate (*n* = 66, r = −0.221, *p* = 0.091). A scatterplot of the relationships between C_min_ and ECMO duration on sampling day was shown in Figure 5.

## Discussion

This is the first retrospective study to demonstrate the significant impact of ECMO on voriconazole exposure. In addition, our study also identified other important clinical factors and drug interactions that predict voriconazole exposure in critically ill patients. These findings help intensive care unit physicians better adapt the dose of voriconazole while treating these patients.

Currently, descriptions of the sequestration of voriconazole on ECMO circuits are mainly limited to *ex vivo* studies and case reports (Mehta et al., 2007; Brüggemann et al., 2008; Ruiz et al., 2009; Spriet et al., 2009; Winiszewski et al., 2018; Cies et al., 2020; Raffaeli et al., 2020; Zhang et al., 2021b; Mathieu et al., 2021; Peterson et al., 2021). Based on these published studies, a conventional dose of voriconazole does not seem to guarantee its therapeutic concentrations in ECMO patients, and it appears necessary to use a higher dose empirically. However, there are some limitations in these studies, which reduce the reliability of this insight. For example, *ex vivo* studies might not truly reflect the processes of drugs *in vivo*, and critical illness-related physiological changes, such as systemic inflammatory response, hypoalbuminemia, renal and/or hepatic function impairment were not taken into account. In addition, *ex vivo* studies only assessed losses at 24 h and therefore did not reflect possible changes in long-term ECMO support (Mehta et al., 2007; Cies et al., 2020; Raffaeli et al., 2020; Zhang et al., 2021b). With regard to case reports, the limitation section of these studies often mentioned some non-ECMO-related causes that might contribute to the low exposure of voriconazole, such as the CYP2C19 genotype status of the patients in their study (Brüggemann et al., 2008; Ruiz et al., 2009; Spriet et al., 2009; Peterson et al., 2021). In contrast to previously published *ex vivo* studies and case reports, a recently published large retrospective analysis found no significant PK changes in voriconazole during ECMO support (Van Daele et al., 2021). Therefore, further studies are needed to assess the real impact of ECMO on voriconazole exposure.

Our study reported for the first time that ECMO is an independent risk factor for inadequate voriconazole exposure. Numerous studies have shown that ECMO is associated with significant PK alterations, most important of which are circuit sequestration, increased volume of distribution (Vd) and altered clearance (CL) (Gomez et al., 2022). From a PK point of view, the attached extracorporeal circuit with a large surface area can sequester drugs during drug transit, as well as modulate their Vd and CL, which is an important factor leading to the PK changes. On the basis of *ex-vivo* and *in-vivo* animal data (Shekar et al., 2012; Shekar et al., 2015a; Shekar et al., 2015b), lipophilic and highly protein-bound drugs are more prone to circuit drug loss. Therefore, it can be expected that voriconazole with these properties will sequester significantly in the ECMO circuits. Our study provides an important basis for this seemingly “well-known” phenomenon and contributes to optimize the clinical application of voriconazole in this patient population. However, a retrospective analysis by Daele in 69 patients with 337 trough concentrations did not find the association between ECMO and voriconazole exposure (Van Daele et al., 2021). In our opinion, this may be attributed to the extensive intra-individual variability between the samples in their study. Interestingly, we also found for the first time that ECMO blood flow rate seemed to be negatively correlated with voriconazole exposure, which might suggest the impact of extracorporeal circulation on voriconazole PK. Admittedly, this correlation may merely indicate hyperdynamic circulation with increased CL in critical conditions, where a higher ECMO blood flow rate is often required to maintain adequate systemic oxygenation. This condition is not uncommon in septic patients and could lead to increased CL of certain drugs (Sime et al., 2015). The impact of ECMO itself and its related factors, such as blood flow rate and duration of ECMO on voriconazole exposure, warrants further investigation in the future.

Voriconazole is mainly metabolized by the drug-metabolizing enzyme cytochrome P450 (CYP) 2C19, and to a lesser extent by CYP3A4 and CYP2C9 (Zhong et al., 2018). Theoretically, the concomitant use of inducers or inhibitors of these enzymes should impact voriconazole exposure. The drug-drug interaction between voriconazole and glucocorticoids, leading to the reduced voriconazole concentration has been suggested by a number of studies previously and has been proposed to be the result of CYP induction by glucocorticoids (Dolton et al., 2012; Jia et al., 2021; Mafuru et al., 2021). Dolton et al (2012) also found that coadministration of methylprednisolone and dexamethasone reduced the voriconazole concentration to a greater extent than prednisone or prednisolone. In our study, 48.5% of patients were treated with glucocorticoids, and most of them were methylprednisolone. Our data supported such an interaction. In the present study, 65.2% of patients received glucocorticoids while receiving ECMO support, which is not uncommon in the clinic (Yu et al., 2021). As discussed above, ECMO is associated with a significant reduction of voriconazole concentration, and physicians should be aware of the possible synergistic effect of both on reducing voriconazole exposure. Another drug-drug interaction, between voriconazole and the CYP inhibitor PPIs, resulting in an increase in voriconazole concentration, is also often reported (Dolton et al., 2012; Gautier-Veyret et al., 2015; Tian et al., 2021). However, in our study, univariate analysis showed that concomitant treatment with PPIs was associated with lower voriconazole concentrations. In our opinion, this conflicting result may be related to the wider use of PPIs in patients with ECMO support.

Similar to the results of many previous studies (Dolton et al., 2012; Zhao et al., 2021a; Zhao Y. C et al., 2021), the linear regression model found that voriconazole trough concentration is significantly affected by the dose before sampling. The administration dosage is the major factor contributing to the highly variable concentrations of voriconazole. Currently, there are no dose recommendations for patients receiving ECMO support, which means that an empirical regimen is a common choice in clinical practice. According to the medication label of voriconazole, the common medication regimen is 200 mg, q12h, which was used by most patients in our hospital, and it is also adopted by many other centers in the world (Zhang et al., 2021a). Our results suggest that conventional dosing in ECMO patients is at risk of under-dosing. On the other hand, although (Van Daele et al., 2021) used a higher dose of voriconazole (8.3 [6.6–10.9] mg/kg in their study), subtherapeutic concentrations (< 2 mg/L) were observed in 56% of the samples during ECMO, which is very similar to our result (51.5%). This emphasizes the effect of non-dose factors on voriconazole exposure. Indeed, according to the standardized coefficients of the final model, the contribution of dose before sampling to voriconazole concentrations was much smaller than ECMO and drug-drug interaction, without collinearity with each other (variance inflation factor < 5).

Finally, our study found a significant association between SOFA score and voriconazole concentrations. The SOFA score is based on the degree of dysfunction of six organ systems including respiratory, circulation, hepatic, coagulation, renal, and neurological systems (Lambden et al., 2019). Among organ dysfunction, hepatic impairment may occur in patients with high SOFA scores. Since the metabolism of voriconazole is regulated by liver enzymes (Zhong et al., 2018), its overexposure may occur in patients with liver damage. Published studies demonstrated that reduced voriconazole elimination is significantly associated with impaired liver function, as indicated by elevated alanine transaminase (Zhao et al., 2021b), aspartate transaminase (Li et al., 2017), direct bilirubin (Zhao Y. C et al., 2021) and international normalized ratio levels (Zhao et al., 2021a). On the other hand, higher SOFA scores also tend to represent lower platelet counts. According to population PK studies, low platelet counts were correlated with significantly reduced voriconazole CL (Tang et al., 2019). This may also partly explain the effect of SOFA score on voriconazole exposure.

We acknowledge several limitations to our study. First, we did not know the CYP2C19 genotype status of the enrolled patients, and therefore its influence on voriconazole C_min_ was not measured. However, it has been noted that the variant alleles with decreased enzyme function (CYP2C19 *2 and CYP2C19 *3) are highly distributed in East Asians, while the frequency of CYP2C19*17 which confers increased enzyme activity is less than 2% in this population, indicating that Chinese people are susceptible to supratherapeutic concentrations due to slow metabolism (Hu et al., 2012). We believe that this background highlights the impact of ECMO on voriconazole exposure. Second, this study was conducted in a single center and on a small number of patients, and it might restrict the ability to generate other statistically significant results. Finally, this is a retrospective study, although we attempted to reduce potential confounding factors, there are still some inevitable confounding factors, such as the differences of individual indicators on the baseline. Therefore, further well-designed and prospective population pharmacokinetic-pharmacodynamic studies for ECMO patients are necessary to gain more and better evidence and recommend appropriate dosing regimens for this patient population.

## Conclusion

This retrospective study in critically ill patients with or without ECMO support infer some novel clinical implications for voriconazole therapy. In addition to the known drug interactions, this study demonstrated for the first time that ECMO is a significant covariable affecting voriconazole exposure. Furthermore, the study also showed that an increase in SOFA score was associated with an increase in voriconazole trough concentration. In light of the highly variable trough levels observed and the existence of various factors that significantly influenced its exposure, TDM of voriconazole remains important, especially in ECMO patients often presenting with subtherapeutic exposure.

## Data Availability

The original contributions presented in the study are included in the article/Supplementary Material, further inquiries can be directed to the corresponding author.

## References

[B1] Ashbee H. R. Barnes R. A. Johnson E. M. Richardson M. D. Gorton R. Hope W. W. (2014). Therapeutic drug monitoring (TDM) of antifungal agents: guidelines from the British society for medical mycology. J. Antimicrob. Chemother. 69 (5), 1162–1176. 10.1093/jac/dkt508 24379304 PMC3977608

[B2] Bartoletti M. Pascale R. Cricca M. Rinaldi M. Maccaro A. Bussini L. (2021). Epidemiology of invasive pulmonary aspergillosis among intubated patients with COVID-19: a prospective study. Clin. Infect. Dis. 73 (11), e3606–e3614. 10.1093/cid/ciaa1065 32719848 PMC7454393

[B3] Bassetti M. Bouza E. (2017). Invasive mould infections in the ICU setting: complexities and solutions. J. Antimicrob. Chemother. 72, i39–i47. 10.1093/jac/dkx032 28355466

[B4] Blanco-Dorado S. Maroñas O. Latorre-Pellicer A. Rodríguez Jato M. T. López-Vizcaíno A. Gómez Márquez A. (2020). Impact of CYP2C19 genotype and drug interactions on voriconazole plasma concentrations: a Spain pharmacogenetic-pharmacokinetic prospective multicenter study. Pharmacotherapy 40 (1), 17–25. 10.1002/phar.2351 31782536

[B5] Brodie D. Slutsky A. S. Combes A. (2019). Extracorporeal life support for adults with respiratory failure and related indications: a review. Jama 322 (6), 557–568. 10.1001/jama.2019.9302 31408142

[B6] Brüggemann R. J. Antonius T. Heijst A. Hoogerbrugge P. M. Burger D. M. Warris A. (2008). Therapeutic drug monitoring of voriconazole in a child with invasive aspergillosis requiring extracorporeal membrane oxygenation. Ther. Drug Monit. 30 (6), 643–646. 10.1097/FTD.0b013e3181898b0c 19057370

[B7] Cavayas Y. A. Yusuff H. Porter R. (2018). Fungal infections in adult patients on extracorporeal life support. Crit. Care 22 (1), 98. 10.1186/s13054-018-2023-z 29665838 PMC5905180

[B8] Chamilos G. Luna M. Lewis R. E. Bodey G. P. Chemaly R. Tarrand J. J. (2006). Invasive fungal infections in patients with hematologic malignancies in a tertiary care cancer center: an autopsy study over a 15-year period (1989-2003). Haematologica 91 (7), 986–989. 16757415

[B9] Cies J. J. Moore W. S. 2nd Giliam N. Low T. Marino D. Deacon J. (2020). Oxygenator impact on voriconazole in extracorporeal membrane oxygenation circuits. Perfusion 35 (6), 529–533. 10.1177/0267659120937906 32627659

[B10] De Pauw B. Walsh T. J. Donnelly J. P. Stevens D. A. Edwards J. E. Calandra T. (2008). Revised definitions of invasive fungal disease from the European organization for research and treatment of cancer/invasive fungal infections cooperative group and the national institute of allergy and infectious diseases mycoses study group (EORTC/MSG) consensus group. Clin. Infect. Dis. 46 (12), 1813–1821. 10.1086/588660 18462102 PMC2671227

[B11] Dellière S. Dudoignon E. Fodil S. Voicu S. Collet M. Oillic P. A. (2020). Risk factors associated with COVID-19-associated pulmonary aspergillosis in ICU patients: a French multicentric retrospective cohort. Clin. Microbiol. Infect. 27 (5), 790.e1–790.e5795. 10.1016/j.cmi.2020.12.005 PMC7733556 33316401

[B12] Dolton M. J. McLachlan A. J. (2014). Voriconazole pharmacokinetics and exposure-response relationships: assessing the links between exposure, efficacy and toxicity. Int. J. Antimicrob. Agents 44 (3), 183–193. 10.1016/j.ijantimicag.2014.05.019 25106074

[B13] Dolton M. J. Ray J. E. Chen S. C. Ng K. Pont L. G. McLachlan A. J. (2012). Multicenter study of voriconazole pharmacokinetics and therapeutic drug monitoring. Antimicrob. Agents Chemother. 56 (9), 4793–4799. 10.1128/aac.00626-12 22751544 PMC3421881

[B14] Duceppe M. A. Kanji S. Do A. T. Ruo N. Cavayas Y. A. Albert M. (2021). Pharmacokinetics of commonly used antimicrobials in critically ill adults during extracorporeal membrane oxygenation: a systematic review. Drugs 81 (11), 1307–1329. 10.1007/s40265-021-01557-3 34224115

[B15] Gautier-Veyret E. Fonrose X. Tonini J. Thiebaut-Bertrand A. Bartoli M. Quesada J. L. (2015). Variability of voriconazole plasma concentrations after allogeneic hematopoietic stem cell transplantation: impact of cytochrome p450 polymorphisms and comedications on initial and subsequent trough levels. Antimicrob. Agents Chemother. 59 (4), 2305–2314. 10.1128/aac.04838-14 25645831 PMC4356835

[B16] Gomez F. Veita J. Laudanski K. (2022). Antibiotics and ECMO in the adult population-persistent challenges and practical guides. Antibiot. (Basel) 11 (3), 338. 10.3390/antibiotics11030338 PMC8944696 35326801

[B17] Hu L. M. Dai D. P. Hu G. X. Yang J. F. Xu R. A. Yang L. P. (2012). Genetic polymorphisms and novel allelic variants of CYP2C19 in the Chinese Han population. Pharmacogenomics 13 (14), 1571–1581. 10.2217/pgs.12.141 23148634

[B18] Jia S. J. Gao K. Q. Huang P. H. Guo R. Zuo X. C. Xia Q. (2021). Interactive effects of glucocorticoids and cytochrome P450 polymorphisms on the plasma trough concentrations of voriconazole. Front. Pharmacol. 12, 666296. 10.3389/fphar.2021.666296 34113252 PMC8185288

[B19] Jin H. Wang T. Falcione B. A. Olsen K. M. Chen K. Tang H. (2016). trough concentration of voriconazole and its relationship with efficacy and safety: a systematic review and meta-analysis. J. Antimicrob. Chemother. 71 (7), 1772–1785. 10.1093/jac/dkw045 26968880 PMC4896404

[B20] Lambden S. Laterre P. F. Levy M. M. Francois B. (2019). The SOFA score-development, utility and challenges of accurate assessment in clinical trials. Crit. Care 23 (1), 374. 10.1186/s13054-019-2663-7 31775846 PMC6880479

[B21] Li Z. W. Peng F. H. Yan M. Liang W. Liu X. L. Wu Y. Q. (2017). Impact of CYP2C19 genotype and liver function on voriconazole pharmacokinetics in renal transplant recipients. Ther. Drug Monit. 39 (4), 422–428. 10.1097/ftd.0000000000000425 28604474 PMC5538305

[B22] Mafuru M. Wu S. Mayala H. Msengwa Z. Phillip A. Mgone C. (2021). Analysis of combined effect of CYP2C19 genetic polymorphism and proton pump inhibitors coadministration on trough concentration of voriconazole. Pharmgenomics. Pers. Med. 14, 1379–1389. 10.2147/pgpm.S329662 34754219 PMC8572102

[B23] Mangal N. Hamadeh I. S. Arwood M. J. Cavallari L. H. Samant T. S. Klinker K. P. (2018). Optimization of voriconazole therapy for the treatment of invasive fungal infections in adults. Clin. Pharmacol. Ther. 104 (5), 957–965. 10.1002/cpt.1012 29315506 PMC6037619

[B24] Mathieu A. Thiboutot Z. Ferreira V. Benoit P. Grandjean Lapierre S. Hétu P. O. (2021). Voriconazole sequestration during extracorporeal membrane oxygenation for invasive lung aspergillosis: a case report. Asaio J. 68, e56–e58. 10.1097/mat.0000000000001427 33788798

[B25] Mehta N. M. Halwick D. R. Dodson B. L. Thompson J. E. Arnold J. H. (2007). Potential drug sequestration during extracorporeal membrane oxygenation: results from an *ex vivo* experiment. Intensive Care Med. 33 (6), 1018–1024. 10.1007/s00134-007-0606-2 17404709

[B26] Mitaka H. Kuno T. Takagi H. Patrawalla P. (2021). Incidence and mortality of COVID-19-associated pulmonary aspergillosis: a systematic review and meta-analysis. Mycoses 64 (9), 993–1001. 10.1111/myc.13292 33896063 PMC8251156

[B27] Miyakis S. van Hal S. J. Ray J. Marriott D. (2010). Voriconazole concentrations and outcome of invasive fungal infections. Clin. Microbiol. Infect. 16 (7), 927–933. 10.1111/j.1469-0691.2009.02990.x 19845698

[B28] Patterson T. F. Thompson G. R. 3rd Denning D. W. Fishman J. A. Hadley S. Herbrecht R. (2016). Practice guidelines for the diagnosis and management of aspergillosis: 2016 update by the infectious diseases society of America. Clin. Infect. Dis. 63 (4), e1–e60. 10.1093/cid/ciw326 27365388 PMC4967602

[B29] Peterson E. L. Chittick P. J. Richardson C. L. (2021). Decreasing voriconazole requirement in a patient after extracorporeal membrane oxygenation discontinuation: a case report. Transpl. Infect. Dis. 23 (3), e13545. 10.1111/tid.13545 33316840

[B30] Poth J. M. Schewe J. C. Putensen C. Ehrentraut S. F. (2022). Impact of invasive fungal diseases on survival under veno-venous extracorporeal membrane oxygenation for ARDS. J. Clin. Med. 11 (7), 1940. 10.3390/jcm11071940 35407548 PMC8999842

[B31] Raffaeli G. Cavallaro G. Allegaert K. Koch B. C. P. Mosca F. Tibboel D. (2020). Sequestration of voriconazole and vancomycin into contemporary extracorporeal membrane oxygenation circuits: an *in vitro* study. Front. Pediatr. 8, 468. 10.3389/fped.2020.00468 32974242 PMC7481439

[B32] Ruiz S. Papy E. Da Silva D. Nataf P. Massias L. Wolff M. (2009). Potential voriconazole and caspofungin sequestration during extracorporeal membrane oxygenation. Intensive Care Med. 35 (1), 183–184. 10.1007/s00134-008-1269-3 18795256

[B33] Sanguinetti M. Posteraro B. Beigelman-Aubry C. Lamoth F. Dunet V. Slavin M. (2019). Diagnosis and treatment of invasive fungal infections: looking ahead. J. Antimicrob. Chemother. 74, ii27–ii37. 10.1093/jac/dkz041 31222314

[B34] Schauwvlieghe A. Rijnders B. J. A. Philips N. Verwijs R. Vanderbeke L. Van Tienen C. (2018). Invasive aspergillosis in patients admitted to the intensive care unit with severe influenza: a retrospective cohort study. Lancet. Respir. Med. 6 (10), 782–792. 10.1016/s2213-2600(18)30274-1 30076119

[B35] Shekar K. Roberts J. A. Barnett A. G. Diab S. Wallis S. C. Fung Y. L. (2015a). Can physicochemical properties of antimicrobials be used to predict their pharmacokinetics during extracorporeal membrane oxygenation? Illustrative data from ovine models. Crit. Care 19, 437. 10.1186/s13054-015-1151-y 26667471 PMC4699331

[B36] Shekar K. Roberts J. A. McDonald C. I. Fisquet S. Barnett A. G. Mullany D. V. (2012). Sequestration of drugs in the circuit may lead to therapeutic failure during extracorporeal membrane oxygenation. Crit. Care 16 (5), R194. 10.1186/cc11679 23068416 PMC3682296

[B37] Shekar K. Roberts J. A. McDonald C. I. Ghassabian S. Anstey C. Wallis S. C. (2015b). Protein-bound drugs are prone to sequestration in the extracorporeal membrane oxygenation circuit: results from an *ex vivo* study. Crit. Care 19 (1), 164. 10.1186/s13054-015-0891-z 25888449 PMC4407324

[B38] Shi C. Xiao Y. Mao Y. Wu J. Lin N. (2019). Voriconazole: a review of population pharmacokinetic analyses. Clin. Pharmacokinet. 58 (6), 687–703. 10.1007/s40262-019-00735-7 30687893

[B39] Sime F. B. Udy A. A. Roberts J. A. (2015). Augmented renal clearance in critically ill patients: etiology, definition and implications for beta-lactam dose optimization. Curr. Opin. Pharmacol. 24, 1–6. 10.1016/j.coph.2015.06.002 26119486

[B40] Spriet I. Annaert P. Meersseman P. Hermans G. Meersseman W. Verbesselt R. (2009). Pharmacokinetics of caspofungin and voriconazole in critically ill patients during extracorporeal membrane oxygenation. J. Antimicrob. Chemother. 63 (4), 767–770. 10.1093/jac/dkp026 19218271

[B41] Tang D. Song B. L. Yan M. Zou J. J. Zhang M. Zhou H. Y. (2019). Identifying factors affecting the pharmacokinetics of voriconazole in patients with liver dysfunction: a population pharmacokinetic approach. Basic Clin. Pharmacol. Toxicol. 125 (1), 34–43. 10.1111/bcpt.13208 30715804

[B42] Tian X. Zhang C. Qin Z. Wang D. Yang J. Zhang X. (2021). Impact of CYP2C19 phenotype and drug-drug interactions on voriconazole concentration in pediatric patients. Antimicrob. Agents Chemother. 65 (9), e0020721. 10.1128/aac.00207-21 34152823 PMC8370228

[B43] Tudesq J. J. Peyrony O. Lemiale V. Azoulay E. (2019). Invasive pulmonary aspergillosis in nonimmunocompromised hosts. Semin. Respir. Crit. Care Med. 40 (4), 540–547. 10.1055/s-0039-1696968 31585479

[B44] Van Daele R. Bekkers B. Lindfors M. Broman L. M. Schauwvlieghe A. Rijnders B. (2021). A large retrospective assessment of voriconazole exposure in patients treated with extracorporeal membrane oxygenation. Microorganisms 9 (7), 1543. 10.3390/microorganisms9071543 34361978 PMC8303158

[B45] Verweij P. E. Rijnders B. J. A. Brüggemann R. J. M. Azoulay E. Bassetti M. Blot S. (2020). Review of influenza-associated pulmonary aspergillosis in ICU patients and proposal for a case definition: an expert opinion. Intensive Care Med. 46 (8), 1524–1535. 10.1007/s00134-020-06091-6 32572532 PMC7306567

[B46] Winiszewski H. Rougny A. C. Lagoutte-Renosi J. Millon L. Capellier G. Navellou J. C. (2018). The pharmacokinetic challenge of treating invasive aspergillosis complicating severe influenzae assisted by extracorporeal membrane oxygenation. Crit. Care 22 (1), 355. 10.1186/s13054-018-2285-5 30577863 PMC6303967

[B47] Yu X. Gu S. Li M. Zhan Q. (2021). Awake extracorporeal membrane oxygenation for acute respiratory distress syndrome: which clinical issues should Be taken into consideration. Front. Med. 8, 682526. 10.3389/fmed.2021.682526 PMC8282255 34277659

[B48] Zhang Y. Hou K. Liu F. Luo X. He S. Hu L. (2021a). The influence of CYP2C19 polymorphisms on voriconazole trough concentrations: systematic review and meta-analysis. Mycoses 64 (8), 860–873. 10.1111/myc.13293 33896064

[B49] Zhang Y. Hu H. Zhang Q. Ou Q. Zhou H. Sha T. (2021b). Effects of *ex vivo* extracorporeal membrane oxygenation circuits on sequestration of antimicrobial agents. Front. Med. 8, 748769. 10.3389/fmed.2021.748769 PMC8671752 34926498

[B50] Zhao Y. C. Zou Y. Hou J. J. Xiao C. L. Zhang B. K. Li J. K. (2021). Factors affecting voriconazole trough concentration and optimal maintenance voriconazole dose in Chinese children. Antibiot. (Basel) 10 (12), 1542. 10.3390/antibiotics10121542 PMC8698693 34943754

[B51] Zhao Y. Hou J. Xiao Y. Wang F. Zhang B. Zhang M. (2021a). Predictors of voriconazole trough concentrations in patients with child-pugh class C cirrhosis: a prospective study. Antibiot. (Basel) 10 (9), 1130. 10.3390/antibiotics10091130 PMC8470058 34572712

[B52] Zhao Y. Xiao C. Hou J. Wu J. Xiao Y. Zhang B. (2021b). A large sample retrospective study on the distinction of voriconazole concentration in asian patients from different clinical departments. Pharm. (Basel) 14 (12), 1239. 10.3390/ph14121239 PMC8705093 34959640

[B53] Zhong X. Tong X. Ju Y. Du X. Li Y. (2018). Interpersonal factors in the pharmacokinetics and pharmacodynamics of voriconazole: are CYP2C19 genotypes enough for us to make a clinical decision? Curr. Drug Metab. 19 (14), 1152–1158. 10.2174/1389200219666171227200547 29361899 PMC6635675

